# 2,2′,7,7′-Tetra­bromo-9,9′-spiro­bifluorene toluene hemisolvate

**DOI:** 10.1107/S1600536809021072

**Published:** 2009-06-10

**Authors:** Ailing Guo, Ruitao Zhu

**Affiliations:** a Department of Traditional Chinese Pharmacology, Shanxi University of Traditional Chinese Medicine, Taiyuan 030024, People’s Republic of China; b Department of Chemistry, Taiyuan Normal University, Taiyuan 030031, People’s Republic of China

## Abstract

There are two independent mol­ecules and one toluene solvent mol­ecule in the asymmetric unit of the title compound, C_25_H_12_Br_4_·0.5C_7_H_8_. The dihedral angles between the fluorene ring systems are 85.30 (6) and 84.95 (6)° in the two mol­ecules. The disortions in angles from the ideal *sp* ^3^-hybridization geometry around the tetra­hedral C atoms are due to the strain imposed by the central five-membered ring and steric effects.

## Related literature

For applications of spiro­bifluorene compounds, see: Hagen *et al.* (1997 ▶); Pudzich *et al.* (2006 ▶); Salbeck *et al.* (1997 ▶); Iour *et al.* (1990 ▶). For details of the synthesis, see: Marsitzky & Carter (2001 ▶).
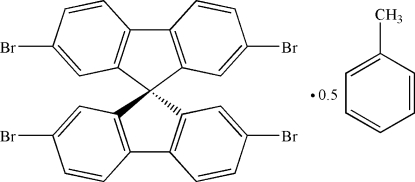


## Experimental

### 

#### Crystal data

C_25_H_12_Br_4_·0.5C_7_H_8_
*M* *_r_* = 678.06
Monoclinic, 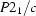

*a* = 14.6593 (18) Å
*b* = 29.549 (4) Å
*c* = 11.3753 (14) Å
β = 96.878 (2)°
*V* = 4891.9 (10) Å^3^
*Z* = 8
Mo *K*α radiation
μ = 6.60 mm^−1^
*T* = 293 K
0.30 × 0.20 × 0.15 mm

#### Data collection

Bruker SMART-CCD diffractometer
Absorption correction: multi-scan (*SADABS*; Sheldrick, 1996 ▶) *T*_min_ = 0.189, *T*_max_ = 0.438 (expected range = 0.161–0.372)
20291 measured reflections
8616 independent reflections
5479 reflections with *I* > 2σ(*I*)
*R*_int_ = 0.045

#### Refinement

*R*[*F* ^2^ > 2σ(*F* ^2^)] = 0.043
*wR*(*F* ^2^) = 0.091
*S* = 1.01
8616 reflections
586 parameters
H-atom parameters constrained
Δρ_max_ = 0.56 e Å^−3^
Δρ_min_ = −0.41 e Å^−3^

### 

Data collection: *SMART* (Bruker, 2000 ▶); cell refinement: *SAINT* (Bruker, 2000 ▶); data reduction: *SAINT*; program(s) used to solve structure: *SHELXS97* (Sheldrick, 2008 ▶); program(s) used to refine structure: *SHELXL97* (Sheldrick, 2008 ▶); molecular graphics: *PLATON* (Spek, 2009 ▶); software used to prepare material for publication: *SHELXTL* (Sheldrick, 2008 ▶).

## Supplementary Material

Crystal structure: contains datablocks I, global. DOI: 10.1107/S1600536809021072/lh2831sup1.cif

Structure factors: contains datablocks I. DOI: 10.1107/S1600536809021072/lh2831Isup2.hkl

Additional supplementary materials:  crystallographic information; 3D view; checkCIF report

## Figures and Tables

**Table 1:** Selected bond angles (°)

C19—C9—C8	112.9 (3)
C19—C9—C21	101.0 (4)
C8—C9—C21	116.8 (4)
C19—C9—C6	113.4 (4)
C8—C9—C6	101.6 (3)
C21—C9—C6	111.6 (3)
C31—C34—C33	100.9 (4)
C31—C34—C46	114.2 (4)
C33—C34—C46	116.9 (4)
C31—C34—C44	113.7 (4)
C33—C34—C44	110.2 (4)
C46—C34—C44	101.5 (4)

**Fig. 1.**
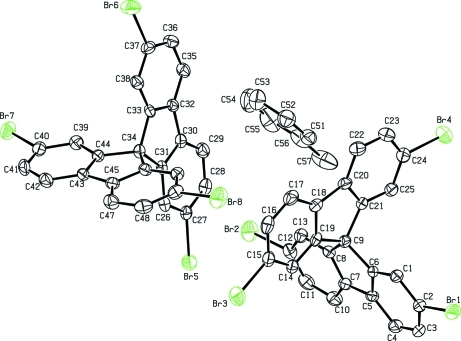
A view of the asymmetric unit of the title compound: displacement ellipsoids are drawn at the 30% probability level. H atoms are not shown.

## supplementary crystallographic information

### Comment

Molecules with a spirobifluorene core have gained wide application in molecular electronics, light-emitting materials production and enantioselective molecular recognition. In addition, macro spiro-organic molecules have attracted interest (Hagen *et al.*, 1997; Salbeck *et al.*, 1997), since they may play a key role in the construction of modern electronic systems and can be used in synthesizing hole transport media which have achieved impressive solar-to-electrical energy conversion efficiencies (James *et al.*, 1990). We are interested in the title compound (TBSBF.0.5(C_7_H_8_), due to its versatility and utility in organic synthesis and herein we report its crystal structure.

The asymmetric unit of the title compound is shown in Fig. 1. The disortions in angles from the ideal [109.5°] sp^3^ hybridization geometry around the tetrahedral C atoms in each molecule [C9 and C34] are due to the strain imposed by the central five-membered ring and from steric effects. The dihedral angles between the fluorene ring systems in each molecule are 85.30 (6) and 84.95 (6)°.

### Experimental

The title compound was synthesized according to the published procedure (Marsitzky & Carter, 2001). To a solution of 9,9'-spirobifluorene (8g, 25.3mmol) in chloroform (100mL) was added bromine (16.6g, 103.7mmol) in 20mL of chloroform (Marsitzky & Carter, 2001). The resulting mixture was stirred overnight at room temperature. The precipitate formed was seperated by filtration and washed with methanol to give the crude target compound. The product, TBSBF, was recrystallized from toluene, giving a yield of 55%.

### Refinement

H atoms were placed in idealized positions and allowed to ride on their respective parent atoms, with C—H = 0.93-0.96 Å and with *U*_iso_(H) = 1.2*U*_eq_(C) or 1.5*U*_eq_(C) for methyl C atoms.

### Crystal data

**Table:**

C_25_H_12_Br_4_·0.5C_7_H_8_	*F*(000) = 2616
*M**_r_* = 678.06	*D*_x_ = 1.841 Mg m^−^^3^
Monoclinic, *P*2_1_/*c*	Mo *K*α radiation, λ = 0.71073 Å
Hall symbol: -P 2ybc	Cell parameters from 3526 reflections
*a* = 14.6593 (18) Å	θ = 2.3–20.5°
*b* = 29.549 (4) Å	µ = 6.60 mm^−^^1^
*c* = 11.3753 (14) Å	*T* = 293 K
β = 96.878 (2)°	Block, colorless
*V* = 4891.9 (10) Å^3^	0.30 × 0.20 × 0.15 mm
*Z* = 8	

### Data collection

**Table:**

Bruker SMART-CCD diffractometer	8616 independent reflections
Radiation source: fine-focus sealed tube	5479 reflections with *I* > 2σ(*I*)
graphite	*R*_int_ = 0.045
ω scans	θ_max_ = 25.0°, θ_min_ = 1.4°
Absorption correction: multi-scan (*SADABS*; Sheldrick, 1996)	*h* = −17→16
*T*_min_ = 0.189, *T*_max_ = 0.438	*k* = −32→35
20291 measured reflections	*l* = −7→13

### Refinement

**Table:**

Refinement on *F*^2^	Primary atom site location: structure-invariant direct methods
Least-squares matrix: full	Secondary atom site location: difference Fourier map
*R*[*F*^2^ > 2σ(*F*^2^)] = 0.043	Hydrogen site location: inferred from neighbouring sites
*wR*(*F*^2^) = 0.091	H-atom parameters constrained
*S* = 1.01	*w* = 1/[σ^2^(*F*_o_^2^) + (0.0144*P*)^2^ + 2.4065*P*] where *P* = (*F*_o_^2^ + 2*F*_c_^2^)/3
8616 reflections	(Δ/σ)_max_ = 0.001
586 parameters	Δρ_max_ = 0.56 e Å^−^^3^
0 restraints	Δρ_min_ = −0.41 e Å^−^^3^

### Special details

**Table:**

Geometry. All e.s.d.'s (except the e.s.d. in the dihedral angle between two l.s. planes) are estimated using the full covariance matrix. The cell e.s.d.'s are taken into account individually in the estimation of e.s.d.'s in distances, angles and torsion angles; correlations between e.s.d.'s in cell parameters are only used when they are defined by crystal symmetry. An approximate (isotropic) treatment of cell e.s.d.'s is used for estimating e.s.d.'s involving l.s. planes.
Refinement. Refinement of *F*^2^ against ALL reflections. The weighted *R*-factor *wR* and goodness of fit *S* are based on *F*^2^, conventional *R*-factors *R* are based on *F*, with *F* set to zero for negative *F*^2^. The threshold expression of *F*^2^ > σ(*F*^2^) is used only for calculating *R*-factors(gt) *etc*. and is not relevant to the choice of reflections for refinement. *R*-factors based on *F*^2^ are statistically about twice as large as those based on *F*, and *R*- factors based on ALL data will be even larger.

### Fractional atomic coordinates and isotropic or equivalent isotropic displacement parameters (Å^2^)

**Table:**

	*x*	*y*	*z*	*U*_iso_*/*U*_eq_	
Br1	−0.17970 (4)	0.057801 (18)	−0.07187 (6)	0.06389 (18)	
Br2	0.41951 (4)	0.06834 (2)	0.54188 (6)	0.0780 (2)	
Br3	0.35211 (4)	0.03188 (2)	−0.13143 (6)	0.0777 (2)	
Br4	−0.03438 (4)	0.24517 (2)	0.36220 (7)	0.0814 (2)	
C1	−0.0114 (3)	0.07959 (15)	0.0645 (4)	0.0405 (12)	
H1A	−0.0104	0.1040	0.0130	0.049*	
C2	−0.0826 (3)	0.04904 (16)	0.0512 (4)	0.0421 (12)	
C3	−0.0845 (3)	0.01259 (16)	0.1269 (5)	0.0498 (14)	
H3A	−0.1329	−0.0080	0.1159	0.060*	
C4	−0.0156 (3)	0.00654 (15)	0.2183 (5)	0.0492 (13)	
H4A	−0.0172	−0.0179	0.2695	0.059*	
C5	0.0568 (3)	0.03736 (14)	0.2340 (4)	0.0375 (11)	
C6	0.0580 (3)	0.07345 (13)	0.1547 (4)	0.0340 (11)	
C7	0.1378 (3)	0.04009 (14)	0.3220 (4)	0.0388 (12)	
C8	0.1896 (3)	0.07777 (14)	0.2979 (4)	0.0347 (11)	
C9	0.1442 (3)	0.10224 (14)	0.1875 (4)	0.0376 (11)	
C10	0.1674 (4)	0.01268 (16)	0.4177 (5)	0.0501 (13)	
H10A	0.1320	−0.0118	0.4364	0.060*	
C11	0.2506 (4)	0.02223 (17)	0.4859 (5)	0.0543 (14)	
H11A	0.2711	0.0042	0.5508	0.065*	
C12	0.3024 (3)	0.05870 (17)	0.4562 (5)	0.0489 (13)	
C13	0.2726 (3)	0.08698 (16)	0.3627 (4)	0.0432 (12)	
H13A	0.3078	0.1116	0.3443	0.052*	
C14	0.2467 (3)	0.06971 (16)	0.0368 (4)	0.0424 (12)	
H14A	0.2388	0.0400	0.0604	0.051*	
C15	0.2998 (3)	0.07970 (17)	−0.0519 (5)	0.0478 (13)	
C16	0.3145 (3)	0.12396 (19)	−0.0839 (5)	0.0560 (15)	
H16A	0.3522	0.1300	−0.1423	0.067*	
C17	0.2743 (3)	0.15924 (18)	−0.0305 (5)	0.0534 (14)	
H17A	0.2847	0.1890	−0.0518	0.064*	
C18	0.2180 (3)	0.14956 (15)	0.0554 (4)	0.0394 (12)	
C19	0.2059 (3)	0.10493 (15)	0.0895 (4)	0.0350 (11)	
C20	0.1634 (3)	0.17866 (15)	0.1246 (4)	0.0425 (12)	
C21	0.1203 (3)	0.15220 (14)	0.2018 (4)	0.0367 (11)	
C22	0.1474 (4)	0.22495 (17)	0.1180 (5)	0.0603 (15)	
H22A	0.1756	0.2428	0.0654	0.072*	
C23	0.0893 (4)	0.24409 (18)	0.1905 (5)	0.0644 (16)	
H23A	0.0789	0.2752	0.1886	0.077*	
C24	0.0467 (3)	0.21714 (18)	0.2652 (5)	0.0534 (14)	
C25	0.0619 (3)	0.17116 (16)	0.2738 (5)	0.0467 (13)	
H25A	0.0336	0.1536	0.3267	0.056*	
Br5	0.57872 (4)	−0.001178 (17)	0.78287 (5)	0.05951 (17)	
Br6	0.81223 (5)	0.319181 (18)	0.53771 (6)	0.0754 (2)	
Br7	1.08306 (4)	0.12112 (2)	0.77786 (6)	0.06736 (18)	
Br8	0.47421 (4)	0.12801 (2)	0.17964 (6)	0.0820 (2)	
C26	0.6546 (3)	0.07355 (16)	0.6731 (4)	0.0422 (12)	
H26A	0.6720	0.0521	0.6201	0.051*	
C27	0.6064 (3)	0.06097 (15)	0.7653 (4)	0.0413 (12)	
C28	0.5793 (3)	0.09210 (18)	0.8438 (4)	0.0479 (13)	
H28A	0.5465	0.0828	0.9047	0.057*	
C29	0.6008 (3)	0.13751 (17)	0.8321 (5)	0.0500 (13)	
H29A	0.5828	0.1588	0.8850	0.060*	
C30	0.6496 (3)	0.15086 (16)	0.7401 (4)	0.0388 (12)	
C31	0.6764 (3)	0.11875 (15)	0.6618 (4)	0.0360 (11)	
C32	0.6816 (3)	0.19535 (15)	0.7053 (4)	0.0366 (11)	
C33	0.7255 (3)	0.19035 (14)	0.6042 (4)	0.0373 (11)	
C34	0.7297 (3)	0.14035 (15)	0.5697 (4)	0.0381 (11)	
C35	0.6764 (3)	0.23790 (17)	0.7552 (5)	0.0494 (13)	
H35A	0.6473	0.2417	0.8229	0.059*	
C36	0.7144 (3)	0.27440 (17)	0.7042 (5)	0.0540 (14)	
H36A	0.7109	0.3031	0.7372	0.065*	
C37	0.7578 (3)	0.26854 (15)	0.6040 (5)	0.0471 (13)	
C38	0.7643 (3)	0.22627 (15)	0.5533 (4)	0.0426 (12)	
H38A	0.7942	0.2224	0.4864	0.051*	
C39	0.8987 (3)	0.12948 (15)	0.6703 (4)	0.0440 (12)	
H39A	0.8871	0.1423	0.7417	0.053*	
C40	0.9858 (3)	0.11444 (16)	0.6533 (5)	0.0487 (13)	
C41	1.0036 (3)	0.09547 (17)	0.5478 (5)	0.0533 (14)	
H41A	1.0627	0.0857	0.5390	0.064*	
C42	0.9350 (3)	0.09092 (15)	0.4558 (5)	0.0507 (14)	
H42A	0.9472	0.0779	0.3849	0.061*	
C43	0.8474 (3)	0.10582 (14)	0.4691 (4)	0.0391 (12)	
C44	0.8301 (3)	0.12461 (14)	0.5775 (4)	0.0366 (11)	
C45	0.7629 (3)	0.10718 (14)	0.3877 (4)	0.0413 (12)	
C46	0.6939 (3)	0.12798 (14)	0.4415 (4)	0.0394 (12)	
C47	0.7454 (4)	0.09201 (16)	0.2721 (5)	0.0526 (14)	
H47A	0.7914	0.0782	0.2353	0.063*	
C48	0.6580 (4)	0.09780 (17)	0.2118 (5)	0.0574 (15)	
H48A	0.6445	0.0874	0.1344	0.069*	
C49	0.5911 (3)	0.11913 (17)	0.2678 (5)	0.0503 (13)	
C50	0.6072 (3)	0.13394 (15)	0.3832 (4)	0.0435 (12)	
H50A	0.5610	0.1475	0.4203	0.052*	
C51	0.3339 (5)	0.1956 (2)	0.5276 (6)	0.0749 (19)	
C52	0.4214 (6)	0.2088 (3)	0.5720 (7)	0.106 (3)	
H52A	0.4446	0.2012	0.6492	0.127*	
C53	0.4744 (6)	0.2331 (3)	0.5035 (10)	0.115 (3)	
H53A	0.5327	0.2424	0.5357	0.138*	
C54	0.4440 (5)	0.2438 (2)	0.3900 (8)	0.095 (2)	
H54A	0.4808	0.2602	0.3442	0.114*	
C55	0.3587 (5)	0.2302 (2)	0.3440 (6)	0.0751 (18)	
H55A	0.3375	0.2366	0.2654	0.090*	
C56	0.3035 (4)	0.20723 (18)	0.4127 (6)	0.0645 (16)	
H56A	0.2443	0.1993	0.3808	0.077*	
C57	0.2722 (5)	0.1698 (2)	0.5987 (6)	0.115 (3)	
H57A	0.3034	0.1643	0.6765	0.173*	
H57B	0.2562	0.1414	0.5606	0.173*	
H57C	0.2174	0.1870	0.6047	0.173*	

### Atomic displacement parameters (Å^2^)

**Table:**

	*U*^11^	*U*^22^	*U*^33^	*U*^12^	*U*^13^	*U*^23^
Br1	0.0470 (3)	0.0561 (3)	0.0828 (5)	−0.0033 (3)	−0.0158 (3)	−0.0017 (3)
Br2	0.0596 (4)	0.0973 (5)	0.0704 (5)	0.0069 (3)	−0.0195 (3)	0.0054 (4)
Br3	0.0682 (4)	0.0893 (5)	0.0820 (5)	0.0022 (3)	0.0355 (4)	−0.0194 (4)
Br4	0.0723 (4)	0.0711 (4)	0.1045 (6)	0.0105 (3)	0.0264 (4)	−0.0221 (4)
C1	0.041 (3)	0.036 (3)	0.046 (3)	−0.003 (2)	0.012 (3)	0.002 (2)
C2	0.037 (3)	0.040 (3)	0.048 (3)	−0.001 (2)	0.002 (2)	−0.006 (3)
C3	0.034 (3)	0.040 (3)	0.075 (4)	−0.008 (2)	0.007 (3)	−0.007 (3)
C4	0.052 (3)	0.038 (3)	0.061 (4)	−0.002 (2)	0.017 (3)	0.005 (3)
C5	0.042 (3)	0.029 (3)	0.044 (3)	−0.004 (2)	0.014 (2)	−0.007 (2)
C6	0.028 (2)	0.029 (2)	0.047 (3)	0.000 (2)	0.011 (2)	−0.002 (2)
C7	0.043 (3)	0.032 (3)	0.042 (3)	0.001 (2)	0.007 (2)	−0.002 (2)
C8	0.038 (3)	0.033 (3)	0.034 (3)	0.004 (2)	0.003 (2)	−0.002 (2)
C9	0.033 (3)	0.034 (3)	0.046 (3)	−0.001 (2)	0.004 (2)	0.006 (2)
C10	0.060 (4)	0.042 (3)	0.049 (3)	0.005 (3)	0.009 (3)	0.010 (3)
C11	0.068 (4)	0.048 (3)	0.046 (3)	0.020 (3)	0.004 (3)	0.012 (3)
C12	0.047 (3)	0.053 (3)	0.046 (3)	0.010 (3)	0.002 (3)	−0.004 (3)
C13	0.042 (3)	0.045 (3)	0.042 (3)	−0.001 (2)	0.006 (3)	0.003 (3)
C14	0.031 (3)	0.044 (3)	0.052 (3)	0.000 (2)	0.003 (2)	0.003 (3)
C15	0.033 (3)	0.059 (3)	0.052 (4)	0.000 (2)	0.005 (3)	−0.005 (3)
C16	0.039 (3)	0.078 (4)	0.052 (4)	−0.005 (3)	0.013 (3)	0.012 (3)
C17	0.049 (3)	0.051 (3)	0.061 (4)	−0.010 (3)	0.008 (3)	0.018 (3)
C18	0.037 (3)	0.041 (3)	0.040 (3)	−0.007 (2)	0.000 (2)	0.005 (2)
C19	0.026 (2)	0.046 (3)	0.032 (3)	−0.005 (2)	−0.002 (2)	0.004 (2)
C20	0.044 (3)	0.034 (3)	0.050 (3)	−0.008 (2)	0.006 (3)	0.003 (2)
C21	0.029 (3)	0.035 (3)	0.045 (3)	−0.002 (2)	0.002 (2)	−0.002 (2)
C22	0.063 (4)	0.044 (3)	0.075 (4)	−0.006 (3)	0.012 (3)	0.010 (3)
C23	0.067 (4)	0.039 (3)	0.086 (5)	0.004 (3)	0.008 (4)	0.001 (3)
C24	0.043 (3)	0.054 (4)	0.063 (4)	−0.001 (3)	0.006 (3)	−0.009 (3)
C25	0.040 (3)	0.042 (3)	0.058 (4)	−0.004 (2)	0.004 (3)	0.005 (3)
Br5	0.0541 (3)	0.0497 (3)	0.0768 (4)	0.0034 (3)	0.0161 (3)	0.0126 (3)
Br6	0.0995 (5)	0.0457 (3)	0.0841 (5)	−0.0111 (3)	0.0232 (4)	0.0011 (3)
Br7	0.0457 (3)	0.0749 (4)	0.0777 (4)	−0.0033 (3)	−0.0082 (3)	0.0151 (3)
Br8	0.0624 (4)	0.1103 (5)	0.0674 (4)	−0.0060 (4)	−0.0166 (3)	−0.0071 (4)
C26	0.033 (3)	0.053 (3)	0.041 (3)	0.010 (2)	0.006 (2)	0.000 (3)
C27	0.029 (3)	0.045 (3)	0.048 (3)	0.003 (2)	−0.002 (2)	0.002 (3)
C28	0.037 (3)	0.067 (4)	0.040 (3)	−0.001 (3)	0.006 (2)	0.001 (3)
C29	0.045 (3)	0.055 (3)	0.051 (3)	0.005 (3)	0.008 (3)	−0.011 (3)
C30	0.030 (3)	0.048 (3)	0.038 (3)	0.004 (2)	0.003 (2)	−0.004 (2)
C31	0.030 (3)	0.042 (3)	0.037 (3)	0.005 (2)	0.008 (2)	0.004 (2)
C32	0.032 (3)	0.039 (3)	0.038 (3)	0.003 (2)	0.001 (2)	−0.008 (2)
C33	0.031 (3)	0.036 (3)	0.045 (3)	0.009 (2)	0.005 (2)	−0.004 (2)
C34	0.032 (3)	0.044 (3)	0.039 (3)	0.002 (2)	0.007 (2)	−0.001 (2)
C35	0.048 (3)	0.053 (3)	0.049 (3)	0.005 (3)	0.014 (3)	−0.014 (3)
C36	0.056 (3)	0.040 (3)	0.065 (4)	0.002 (3)	0.002 (3)	−0.013 (3)
C37	0.046 (3)	0.039 (3)	0.055 (4)	−0.008 (2)	0.006 (3)	0.007 (3)
C38	0.044 (3)	0.043 (3)	0.042 (3)	0.006 (2)	0.007 (2)	−0.006 (3)
C39	0.046 (3)	0.046 (3)	0.041 (3)	0.001 (2)	0.009 (3)	0.002 (2)
C40	0.040 (3)	0.044 (3)	0.062 (4)	0.000 (2)	0.004 (3)	0.014 (3)
C41	0.038 (3)	0.057 (3)	0.069 (4)	0.009 (3)	0.024 (3)	0.006 (3)
C42	0.053 (3)	0.045 (3)	0.058 (4)	0.005 (3)	0.021 (3)	0.001 (3)
C43	0.037 (3)	0.030 (3)	0.052 (3)	0.003 (2)	0.012 (3)	0.002 (2)
C44	0.035 (3)	0.033 (3)	0.042 (3)	0.000 (2)	0.010 (2)	0.002 (2)
C45	0.046 (3)	0.033 (3)	0.046 (3)	−0.001 (2)	0.011 (3)	−0.002 (2)
C46	0.042 (3)	0.033 (3)	0.044 (3)	0.002 (2)	0.008 (3)	0.001 (2)
C47	0.061 (4)	0.054 (3)	0.045 (4)	0.003 (3)	0.013 (3)	−0.011 (3)
C48	0.075 (4)	0.054 (3)	0.042 (3)	−0.010 (3)	0.003 (3)	−0.014 (3)
C49	0.050 (3)	0.056 (3)	0.043 (3)	−0.008 (3)	−0.005 (3)	−0.005 (3)
C50	0.042 (3)	0.042 (3)	0.048 (3)	−0.003 (2)	0.009 (3)	−0.003 (2)
C51	0.103 (6)	0.063 (4)	0.059 (5)	0.028 (4)	0.012 (4)	−0.001 (4)
C52	0.106 (7)	0.133 (7)	0.071 (6)	0.040 (6)	−0.022 (5)	−0.024 (5)
C53	0.073 (6)	0.137 (8)	0.129 (9)	0.011 (5)	−0.014 (6)	−0.030 (7)
C54	0.062 (5)	0.097 (5)	0.124 (7)	0.003 (4)	0.009 (5)	0.001 (5)
C55	0.074 (5)	0.072 (4)	0.077 (5)	0.005 (4)	−0.002 (4)	−0.005 (4)
C56	0.061 (4)	0.054 (4)	0.076 (5)	0.007 (3)	−0.001 (4)	−0.002 (3)
C57	0.186 (9)	0.066 (4)	0.104 (6)	0.023 (5)	0.062 (6)	0.008 (4)

### Geometric parameters (Å, °)

**Table:**

Br1—C2	1.891 (5)	C28—C29	1.388 (6)
Br2—C12	1.891 (5)	C28—H28A	0.9300
Br3—C15	1.889 (5)	C29—C30	1.393 (6)
Br4—C24	1.905 (5)	C29—H29A	0.9300
C1—C6	1.368 (6)	C30—C31	1.390 (6)
C1—C2	1.375 (6)	C30—C32	1.466 (6)
C1—H1A	0.9300	C31—C34	1.520 (6)
C2—C3	1.382 (6)	C32—C35	1.385 (6)
C3—C4	1.372 (7)	C32—C33	1.392 (6)
C3—H3A	0.9300	C33—C38	1.366 (6)
C4—C5	1.394 (6)	C33—C34	1.531 (6)
C4—H4A	0.9300	C34—C46	1.534 (6)
C5—C6	1.398 (6)	C34—C44	1.536 (6)
C5—C7	1.462 (6)	C35—C36	1.374 (6)
C6—C9	1.532 (6)	C35—H35A	0.9300
C7—C10	1.385 (6)	C36—C37	1.381 (7)
C7—C8	1.393 (6)	C36—H36A	0.9300
C8—C13	1.373 (6)	C37—C38	1.384 (6)
C8—C9	1.530 (6)	C38—H38A	0.9300
C9—C19	1.519 (6)	C39—C44	1.375 (6)
C9—C21	1.531 (6)	C39—C40	1.388 (6)
C10—C11	1.394 (7)	C39—H39A	0.9300
C10—H10A	0.9300	C40—C41	1.378 (7)
C11—C12	1.383 (6)	C41—C42	1.369 (7)
C11—H11A	0.9300	C41—H41A	0.9300
C12—C13	1.382 (6)	C42—C43	1.383 (6)
C13—H13A	0.9300	C42—H42A	0.9300
C14—C19	1.374 (6)	C43—C44	1.402 (6)
C14—C15	1.378 (6)	C43—C45	1.455 (6)
C14—H14A	0.9300	C45—C47	1.384 (7)
C15—C16	1.381 (6)	C45—C46	1.387 (6)
C16—C17	1.375 (6)	C46—C50	1.373 (6)
C16—H16A	0.9300	C47—C48	1.390 (7)
C17—C18	1.383 (6)	C47—H47A	0.9300
C17—H17A	0.9300	C48—C49	1.383 (7)
C18—C19	1.392 (6)	C48—H48A	0.9300
C18—C20	1.466 (6)	C49—C50	1.377 (6)
C20—C21	1.383 (6)	C50—H50A	0.9300
C20—C22	1.388 (6)	C51—C56	1.374 (8)
C21—C25	1.373 (6)	C51—C52	1.378 (9)
C22—C23	1.376 (7)	C51—C57	1.493 (8)
C22—H22A	0.9300	C52—C53	1.368 (10)
C23—C24	1.369 (7)	C52—H52A	0.9300
C23—H23A	0.9300	C53—C54	1.353 (10)
C24—C25	1.378 (6)	C53—H53A	0.9300
C25—H25A	0.9300	C54—C55	1.357 (8)
Br5—C27	1.896 (4)	C54—H54A	0.9300
Br6—C37	1.894 (4)	C55—C56	1.370 (8)
Br7—C40	1.896 (5)	C55—H55A	0.9300
Br8—C49	1.898 (5)	C56—H56A	0.9300
C26—C31	1.383 (6)	C57—H57A	0.9600
C26—C27	1.384 (6)	C57—H57B	0.9600
C26—H26A	0.9300	C57—H57C	0.9600
C27—C28	1.374 (6)		
			
C6—C1—C2	118.8 (4)	C31—C30—C29	119.9 (4)
C6—C1—H1A	120.6	C31—C30—C32	108.3 (4)
C2—C1—H1A	120.6	C29—C30—C32	131.8 (4)
C1—C2—C3	121.0 (5)	C26—C31—C30	120.9 (4)
C1—C2—Br1	119.1 (4)	C26—C31—C34	127.8 (4)
C3—C2—Br1	119.9 (4)	C30—C31—C34	111.3 (4)
C4—C3—C2	120.4 (4)	C35—C32—C33	119.2 (4)
C4—C3—H3A	119.8	C35—C32—C30	131.9 (4)
C2—C3—H3A	119.8	C33—C32—C30	108.8 (4)
C3—C4—C5	119.5 (5)	C38—C33—C32	121.7 (4)
C3—C4—H4A	120.3	C38—C33—C34	127.6 (4)
C5—C4—H4A	120.3	C32—C33—C34	110.5 (4)
C4—C5—C6	119.0 (5)	C31—C34—C33	100.9 (4)
C4—C5—C7	131.9 (5)	C31—C34—C46	114.2 (4)
C6—C5—C7	109.1 (4)	C33—C34—C46	116.9 (4)
C1—C6—C5	121.3 (4)	C31—C34—C44	113.7 (4)
C1—C6—C9	128.8 (4)	C33—C34—C44	110.2 (4)
C5—C6—C9	109.9 (4)	C46—C34—C44	101.5 (4)
C10—C7—C8	119.8 (5)	C36—C35—C32	119.6 (5)
C10—C7—C5	131.2 (4)	C36—C35—H35A	120.2
C8—C7—C5	109.0 (4)	C32—C35—H35A	120.2
C13—C8—C7	121.4 (4)	C35—C36—C37	120.0 (4)
C13—C8—C9	128.1 (4)	C35—C36—H36A	120.0
C7—C8—C9	110.3 (4)	C37—C36—H36A	120.0
C19—C9—C8	112.9 (3)	C36—C37—C38	121.2 (4)
C19—C9—C21	101.0 (4)	C36—C37—Br6	119.2 (4)
C8—C9—C21	116.8 (4)	C38—C37—Br6	119.6 (4)
C19—C9—C6	113.4 (4)	C33—C38—C37	118.2 (4)
C8—C9—C6	101.6 (3)	C33—C38—H38A	120.9
C21—C9—C6	111.6 (3)	C37—C38—H38A	120.9
C7—C10—C11	119.3 (5)	C44—C39—C40	117.4 (5)
C7—C10—H10A	120.3	C44—C39—H39A	121.3
C11—C10—H10A	120.3	C40—C39—H39A	121.3
C12—C11—C10	119.4 (5)	C41—C40—C39	121.7 (5)
C12—C11—H11A	120.3	C41—C40—Br7	119.3 (4)
C10—C11—H11A	120.3	C39—C40—Br7	119.0 (4)
C13—C12—C11	121.8 (5)	C42—C41—C40	120.5 (5)
C13—C12—Br2	119.3 (4)	C42—C41—H41A	119.7
C11—C12—Br2	118.9 (4)	C40—C41—H41A	119.7
C8—C13—C12	118.2 (4)	C41—C42—C43	119.5 (5)
C8—C13—H13A	120.9	C41—C42—H42A	120.3
C12—C13—H13A	120.9	C43—C42—H42A	120.3
C19—C14—C15	118.1 (4)	C42—C43—C44	119.3 (5)
C19—C14—H14A	120.9	C42—C43—C45	132.0 (5)
C15—C14—H14A	120.9	C44—C43—C45	108.7 (4)
C14—C15—C16	121.0 (5)	C39—C44—C43	121.6 (4)
C14—C15—Br3	119.2 (4)	C39—C44—C34	128.3 (4)
C16—C15—Br3	119.8 (4)	C43—C44—C34	110.0 (4)
C17—C16—C15	120.9 (5)	C47—C45—C46	120.0 (5)
C17—C16—H16A	119.6	C47—C45—C43	130.0 (5)
C15—C16—H16A	119.6	C46—C45—C43	109.9 (4)
C16—C17—C18	118.6 (5)	C50—C46—C45	121.7 (5)
C16—C17—H17A	120.7	C50—C46—C34	128.3 (4)
C18—C17—H17A	120.7	C45—C46—C34	109.9 (4)
C17—C18—C19	120.0 (5)	C45—C47—C48	119.0 (5)
C17—C18—C20	131.9 (4)	C45—C47—H47A	120.5
C19—C18—C20	108.1 (4)	C48—C47—H47A	120.5
C14—C19—C18	121.3 (4)	C49—C48—C47	119.4 (5)
C14—C19—C9	127.5 (4)	C49—C48—H48A	120.3
C18—C19—C9	111.2 (4)	C47—C48—H48A	120.3
C21—C20—C22	120.3 (5)	C50—C49—C48	122.3 (5)
C21—C20—C18	109.2 (4)	C50—C49—Br8	120.0 (4)
C22—C20—C18	130.4 (5)	C48—C49—Br8	117.7 (4)
C25—C21—C20	120.8 (4)	C46—C50—C49	117.5 (5)
C25—C21—C9	128.6 (4)	C46—C50—H50A	121.2
C20—C21—C9	110.5 (4)	C49—C50—H50A	121.2
C23—C22—C20	119.0 (5)	C56—C51—C52	117.3 (7)
C23—C22—H22A	120.5	C56—C51—C57	119.7 (7)
C20—C22—H22A	120.5	C52—C51—C57	123.0 (7)
C24—C23—C22	119.6 (5)	C53—C52—C51	120.5 (8)
C24—C23—H23A	120.2	C53—C52—H52A	119.7
C22—C23—H23A	120.2	C51—C52—H52A	119.7
C23—C24—C25	122.4 (5)	C54—C53—C52	121.4 (8)
C23—C24—Br4	118.0 (4)	C54—C53—H53A	119.3
C25—C24—Br4	119.6 (4)	C52—C53—H53A	119.3
C21—C25—C24	117.8 (5)	C53—C54—C55	118.8 (8)
C21—C25—H25A	121.1	C53—C54—H54A	120.6
C24—C25—H25A	121.1	C55—C54—H54A	120.6
C31—C26—C27	118.3 (4)	C54—C55—C56	120.4 (7)
C31—C26—H26A	120.8	C54—C55—H55A	119.8
C27—C26—H26A	120.8	C56—C55—H55A	119.8
C28—C27—C26	121.8 (4)	C55—C56—C51	121.4 (6)
C28—C27—Br5	119.8 (4)	C55—C56—H56A	119.3
C26—C27—Br5	118.4 (4)	C51—C56—H56A	119.3
C27—C28—C29	119.8 (5)	C51—C57—H57A	109.5
C27—C28—H28A	120.1	C51—C57—H57B	109.5
C29—C28—H28A	120.1	H57A—C57—H57B	109.5
C28—C29—C30	119.3 (5)	C51—C57—H57C	109.5
C28—C29—H29A	120.4	H57A—C57—H57C	109.5
C30—C29—H29A	120.4	H57B—C57—H57C	109.5
